# Supplementary material for: Severe osteoarthritis in aged PANX3 knockout mice: implications for a novel primary osteoarthritis model
Source: JBMR Plus. 2025 Apr 7;9(6):ziaf057. doi: 10.1093/jbmrpl/ziaf057 (PMC12083983; doi:10.1093/jbmrpl/ziaf057)
Supplement: SupplementaryFigures1-4_WakefieldTang_ziaf057 [file supplementaryfigures1-4_wakefieldtang_ziaf057.pdf]

Suppl. Fig. 1

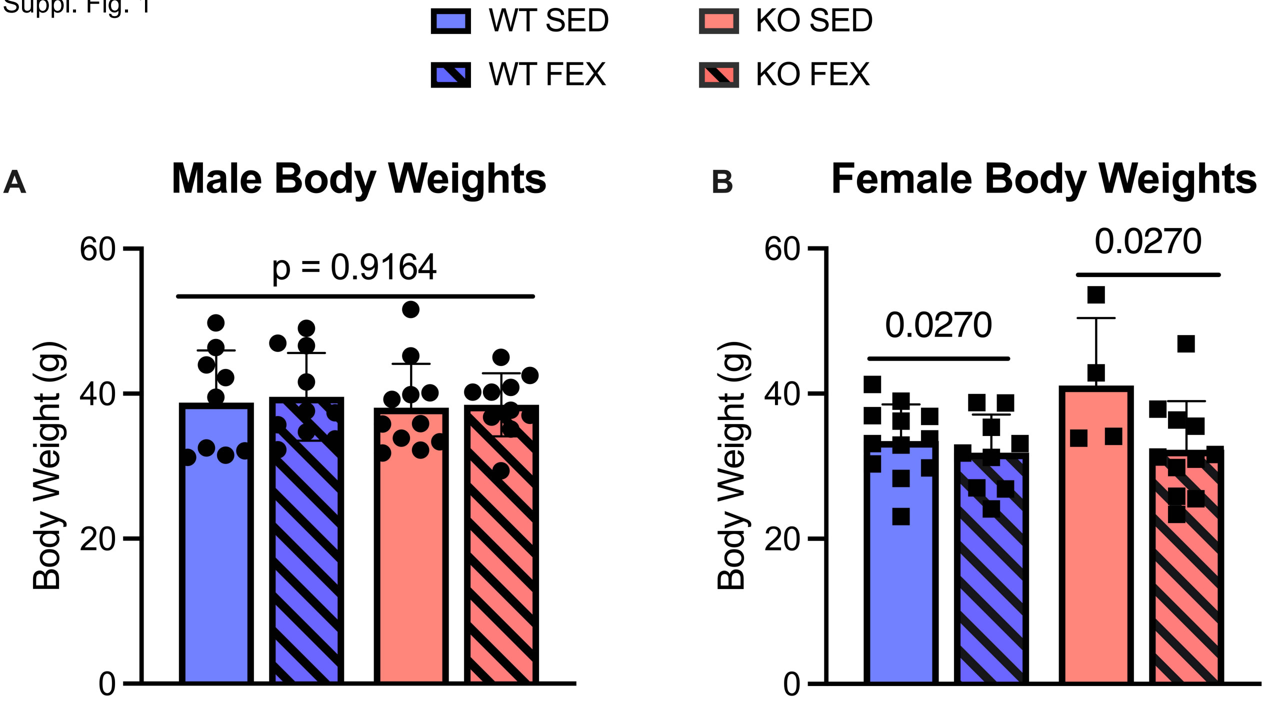
WT SED
KO SED
WT FEX
KO FEX
A
Male Body Weights
p = 0.9164
Body Weight (g)
60
40
20
0
B
Female Body Weights
0.0270
0.0270
Body Weight (g)
60
40
20
0

Suppl. Fig. 2

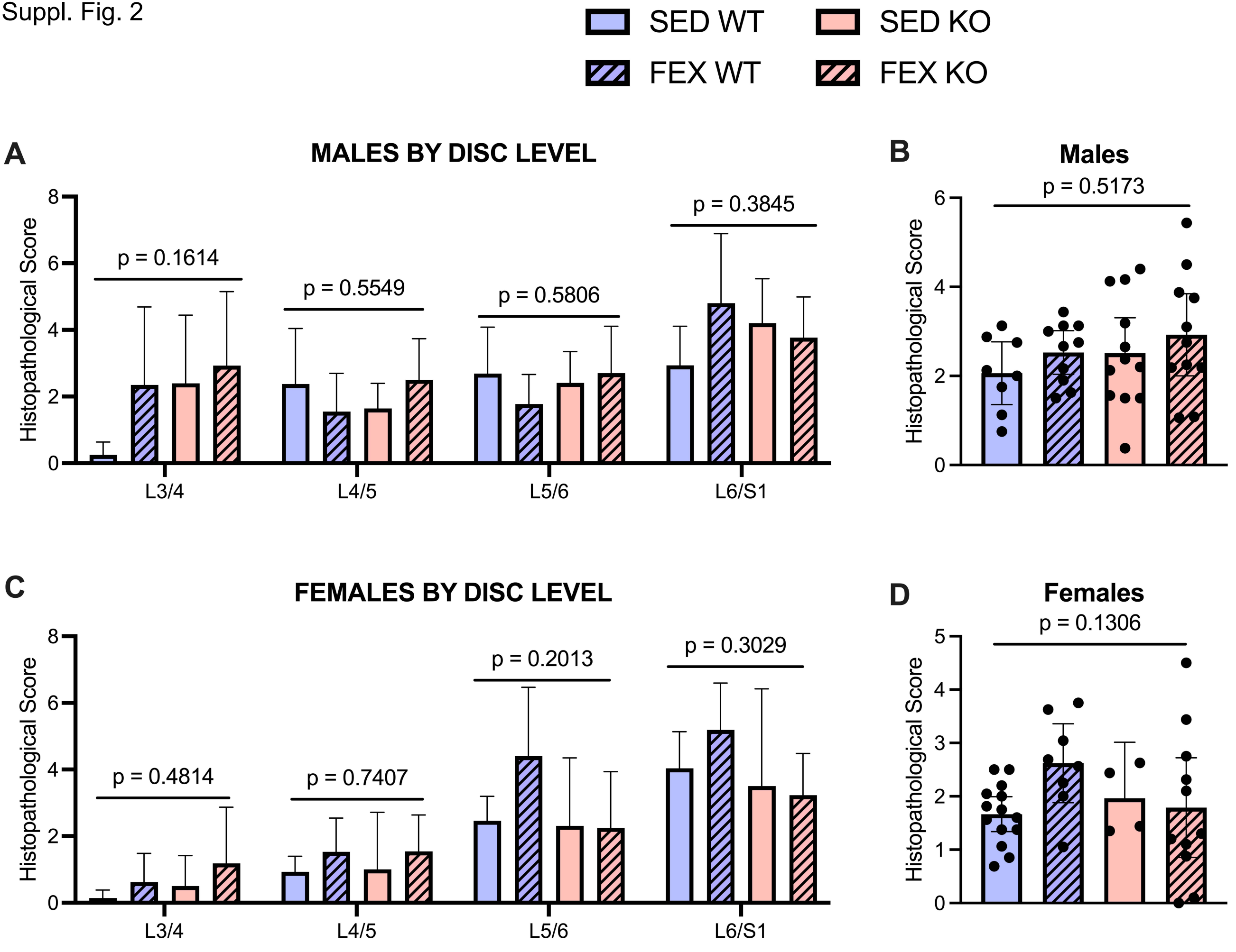
SED WT
SED KO
FEX WT
FEX KO
A
MALES BY DISC LEVEL
Histopathological Score
p = 0.1614
p = 0.5549
p = 0.5806
p = 0.3845
L3/4
L4/5
L5/6
L6/S1
B
Males
p = 0.5173
C
FEMALES BY DISC LEVEL
p = 0.4814
p = 0.7407
p = 0.2013
p = 0.3029
D
Females
p = 0.1306

Suppl. Fig. 3

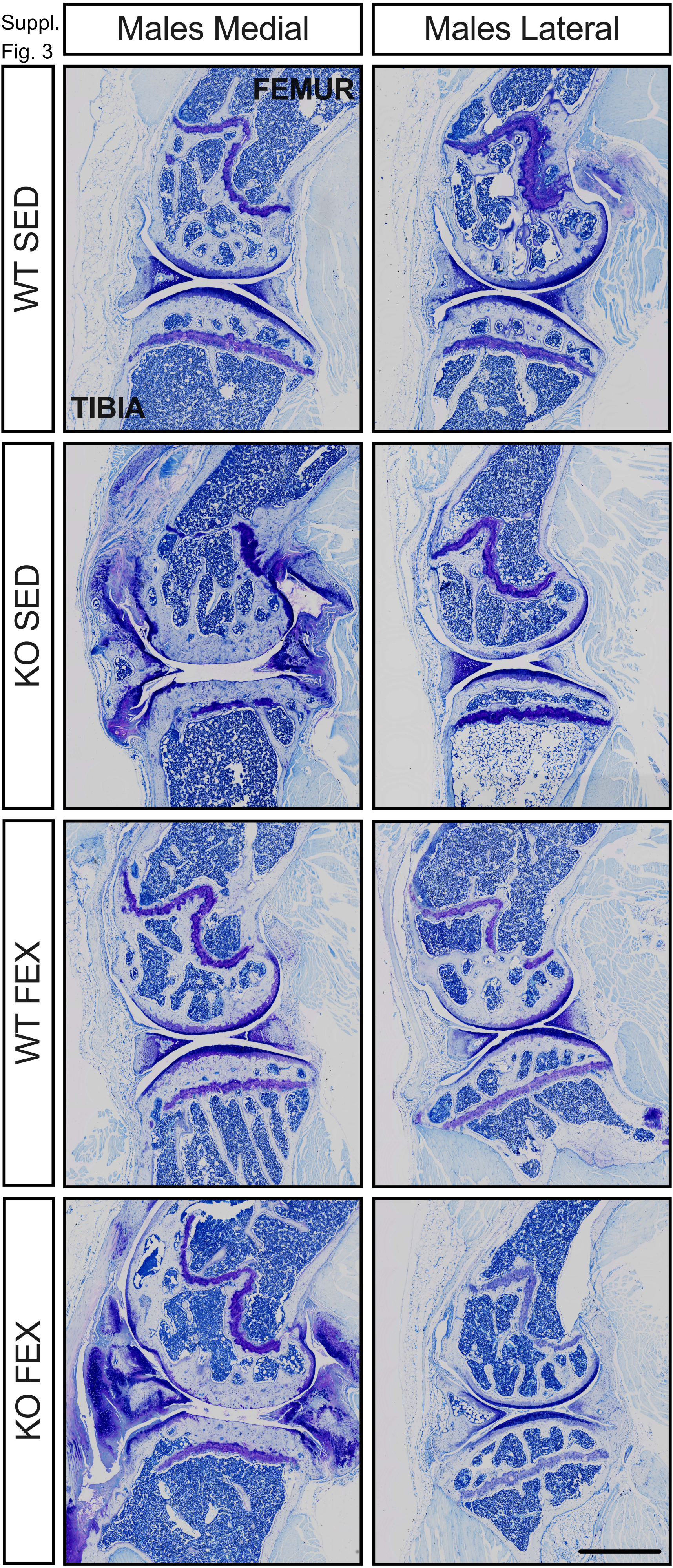
Males Medial
Males Lateral
WT SED
KO SED
WT FEX
KO FEX
FEMUR
TIBIA

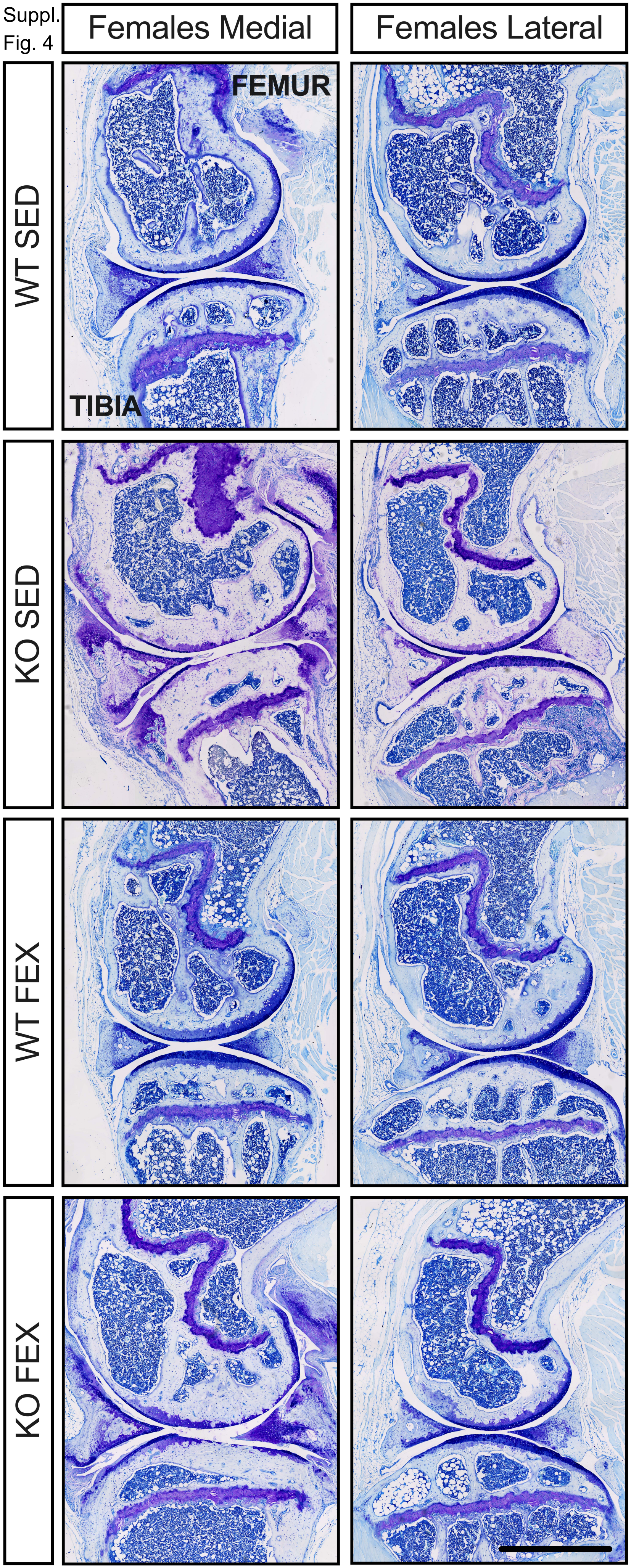
Suppl. Fig. 4
Females Medial
Females Lateral
WT SED
KO SED
WT FEX
KO FEX
FEMUR
TIBIA
