# Supplementary material for: Severe osteoarthritis in aged PANX3 knockout mice: implications for a novel primary osteoarthritis model
Source: JBMR Plus. 2025 Apr 7;9(6):ziaf057. doi: 10.1093/jbmrpl/ziaf057 (PMC12083983; doi:10.1093/jbmrpl/ziaf057)
Supplement: SupplementaryTables_WakefieldTang2025_ziaf057 [file supplementarytables_wakefieldtang2025_ziaf057.docx]

**Supplementary Table 1. Comprehensive list of male OARSI scoring Dunn’s Multiple Comparisons statistical values.**

**Male Tibia Medial OARSI Scores Multiple Comparisons**

| Dunn’s Multiple Comparison | Mean Rank | Z-Value | Adjust P Value |
| --- | --- | --- | --- |
| WT SED vs WT FEX | -8.200 | 1.769 | 0.4613 |
| WT SED vs KO SED | -13.15 | 2.837 | 0.0273 |
| WT SED vs KO FEX | -11.22 | 2.363 | 0.1086 |
| WT FEX vs KO SED | -4.950 | 1.133 | >0.9999 |
| WT FEX vs KO FEX | -3.022 | 0.6731 | >0.9999 |
| KO SED vs KO FEX | 1.928 | 0.4294 | >0.9999 |

**Male Femur Medial OARSI Scores Multiple Comparisons**

| Dunn’s Multiple Comparison | Mean Rank | Z-Value | Adjust P Value |
| --- | --- | --- | --- |
| WT SED vs WT FEX | -0.5333 | 0.1098 | >0.9999 |
| WT SED vs KO SED | -10.33 | 2.011 | 0.2661 |
| WT SED vs KO FEX | -5.242 | 1.103 | >0.9999 |
| WT FEX vs KO SED | -9.800 | 1.954 | 0.3046 |
| WT FEX vs KO FEX | -4.709 | 1.019 | >0.9999 |
| KO SED vs KO FEX | 5.091 | 1.036 | >0.9999 |

**Male Tibia Lateral OARSI Scores Multiple Comparisons**

| Dunn’s Multiple Comparison | Mean Rank | Z-Value | Adjust P Value |
| --- | --- | --- | --- |
| WT SED vs WT FEX | -7.311 | 1.574 | 0.6929 |
| WT SED vs KO SED | -4.011 | 0.8636 | >0.9999 |
| WT SED vs KO FEX | -11.80 | 2.402 | 0.0979 |
| WT FEX vs KO SED | 3.300 | 0.7299 | >0.9999 |
| WT FEX vs KO FEX | -4.488 | 0.9358 | >0.9999 |
| KO SED vs KO FEX | -7.788 | 1.624 | 0.6262 |

**Male Femur Lateral OARSI Scores Multiple Comparisons**

| Dunn’s Multiple Comparison | Mean Rank | Z-Value | Adjust P Value |
| --- | --- | --- | --- |
| WT SED vs WT FEX | -0.7556 | 0.1611 | >0.9999 |
| WT SED vs KO SED | -3.743 | 0.7547 | >0.9999 |
| WT SED vs KO FEX | -5.419 | 1.181 | >0.9999 |
| WT FEX vs KO SED | -2.988 | 0.6170 | >0.9999 |
| WT FEX vs KO FEX | -4.664 | 1.046 | >0.9999 |
| KO SED vs KO FEX | -1.676 | 0.3534 | >0.9999 |

**Supplementary Table 2. Comprehensive list of female OARSI scoring Dunn’s Multiple Comparisons statistical values.**

**Female Tibia Medial OARSI Scores Multiple Comparisons**

| Dunn’s Multiple Comparison | Mean Rank | Z-Value | Adjust P Value |
| --- | --- | --- | --- |
| WT SED vs WT FEX | -5.847 | 1.272 | >0.9999 |
| WT SED vs KO SED | -10.09 | 1.819 | 0.4133 |
| WT SED vs KO FEX | -11.79 | 2.711 | 0.0403 |
| WT FEX vs KO SED | -4.244 | 0.7302 | >0.9999 |
| WT FEX vs KO FEX | -5.944 | 1.269 | >0.9999 |
| KO SED vs KO FEX | -1.700 | 0.3024 | >0.9999 |

**Female Femur Medial OARSI Scores Multiple Comparisons**

| Dunn’s Multiple Comparison | Mean Rank | Z-Value | Adjust P Value |
| --- | --- | --- | --- |
| WT SED vs WT FEX | -9.222 | 1.969 | 0.2935 |
| WT SED vs KO SED | -9.700 | 1.716 | 0.5170 |
| WT SED vs KO FEX | -14.95 | 3.374 | 0.0045 |
| WT FEX vs KO SED | -0.4778 | 0.08066 | >0.9999 |
| WT FEX vs KO FEX | -5.732 | 1.201 | >0.9999 |
| KO SED vs KO FEX | -5.255 | 0.9174 | >0.9999 |

**Female Tibia Lateral OARSI Scores Multiple Comparisons**

| Dunn’s Multiple Comparison | Mean Rank | Z-Value | Adjust P Value |
| --- | --- | --- | --- |
| WT SED vs WT FEX | 0.02778 | 0.006005 | >0.9999 |
| WT SED vs KO SED | -7.683 | 1.376 | >0.9999 |
| WT SED vs KO FEX | -10.27 | 2.344 | 0.1144 |
| WT FEX vs KO SED | -7.711 | 1.318 | >0.9999 |
| WT FEX vs KO FEX | -10.29 | 2.183 | 0.1743 |
| KO SED vs KO FEX | -2.582 | 0.4563 | >0.9999 |

**Female Femur Lateral OARSI Scores Multiple Comparisons**

| Dunn’s Multiple Comparison | Mean Rank | Z-Value | Adjust P Value |
| --- | --- | --- | --- |
| WT SED vs WT FEX | -7.505 | 1.677 | 0.5607 |
| WT SED vs KO SED | -7.327 | 1.365 | >0.9999 |
| WT SED vs KO FEX | -9.273 | 2.185 | 0.1735 |
| WT FEX vs KO SED | 0.1778 | 0.03202 | >0.9999 |
| WT FEX vs KO FEX | -1.768 | 0.3951 | >0.9999 |
| KO SED vs KO FEX | -1.945 | 0.3624 | >0.9999 |

**Supplementary Table 3. Comprehensive list of body weight statistical values for both sexes.**

**Male Body Weight Major Effects**

| 2-Way ANOVA | SS (Type III) | MS | F Value | P Value |
| --- | --- | --- | --- | --- |
| Interaction | 0.3780 | 0.3780 | 0.0106 | 0.9184 |
| Genotype | 8.079 | 8.079 | 0.2273 | 0.6364 |
| Activity | 3.442 | 3.442 | 0.0968 | 0.7574 |

**Male Body Weight Multiple Comparisons**

| Tukey’s Multiple Comparison | Predicted Mean Difference | Adjust P Value |
| --- | --- | --- |
| WT SED vs WT FEX | -0.7831 | 0.9917 |
| WT SED vs KO SED | 0.7062 | 0.9935 |
| WT SED vs KO FEX | 0.3129 | 0.9995 |
| WT FEX vs KO SED | 0.1489 | 0.9399 |
| WT FEX vs KO FEX | 1.096 | 0.9762 |
| KO SED vs KO FEX | -0.3933 | 0.9987 |

**Female Body Weight Major Effects**

| 2-Way ANOVA | SS (Type III) | MS | F Value | P Value |
| --- | --- | --- | --- | --- |
| Interaction | 98.66 | 98.66 | 2.621 | 0.1153 |
| Genotype | 120.7 | 120.7 | 3.207 | 0.0828 |
| Activity | 202.4 | 202.4 | 5.376 | 0.0270 |

**Female Body Weight Multiple Comparisons**

| Tukey’s Multiple Comparison | Predicted Mean Difference | Adjust P Value |
| --- | --- | --- |
| WT SED vs WT FEX | 1.571 | 0.9372 |
| WT SED vs KO SED | -7.653 | 0.1562 |
| WT SED vs KO FEX | 1.185 | 0.9666 |
| WT FEX vs KO SED | -9.224 | 0.0788 |
| WT FEX vs KO FEX | -0.3858 | 0.9990 |
| KO SED vs KO FEX | 8.838 | 0.0847 |

**Supplementary Table 4. Comprehensive list of male enthesitis and tendinopathy Dunn’s Multiple Comparisons statistical values.**

**Male Quadriceps Enthesitis Scores Multiple Comparisons**

| Dunn’s Multiple Comparison | Mean Rank | Z-Value | Adjust P Value |
| --- | --- | --- | --- |
| WT SED vs WT FEX | -2.567 | 0.6006 | >0.9999 |
| WT SED vs KO SED | -12.26 | 2.674 | 0.0450 |
| WT SED vs KO FEX | -1.122 | 0.2626 | >0.9999 |
| WT FEX vs KO SED | 9.690 | 2.067 | 0.2322 |
| WT FEX vs KO FEX | 1.444 | 0.3294 | >0.9999 |
| KO SED vs KO FEX | 11.13 | 2.375 | 0.1052 |

**Male Tendinopathy Scores Multiple Comparisons**

| Dunn’s Multiple Comparison | Mean Rank | Z-Value | Adjust P Value |
| --- | --- | --- | --- |
| WT SED vs WT FEX | -7.900 | 1.782 | 0.4484 |
| WT SED vs KO SED | -9.338 | 2.040 | 0.2479 |
| WT SED vs KO FEX | -7.400 | 1.669 | 0.5703 |
| WT FEX vs KO SED | 1.438 | 0.3066 | >0.9999 |
| WT FEX vs KO FEX | 0.500 | 0.1099 | >0.9999 |
| KO SED vs KO FEX | 1.938 | 0.4133 | >0.9999 |

**Male Patellar Enthesitis Scores Multiple Comparisons**

| Dunn’s Multiple Comparison | Mean Rank | Z-Value | Adjust P Value |
| --- | --- | --- | --- |
| WT SED vs WT FEX | -3.500 | 0.9055 | >0.9999 |
| WT SED vs KO SED | -11.31 | 2.927 | 0.0205 |
| WT SED vs KO FEX | -6.278 | 1.677 | 0.5615 |
| WT FEX vs KO SED | 7.813 | 1.918 | 0.3310 |
| WT FEX vs KO FEX | -2.778 | 0.7016 | >0.9999 |
| KO SED vs KO FEX | 5.035 | 1.272 | >0.9999 |

**Supplementary Table 5. Comprehensive list of female enthesitis and tendinopathy Dunn’s Multiple Comparisons statistical values.**

**Female Quadriceps Enthesitis Scores Multiple Comparisons**

| Dunn’s Multiple Comparison | Mean Rank | Z-Value | Adjust P Value |
| --- | --- | --- | --- |
| WT SED vs WT FEX | 8.700 | 2.330 | 0.1187 |
| WT SED vs KO SED | 8.700 | 1.745 | 0.4864 |
| WT SED vs KO FEX | -5.800 | 1.666 | 0.5740 |
| WT FEX vs KO SED | 0.000 | 0.000 | >0.9999 |
| WT FEX vs KO FEX | -14.50 | 3.798 | 0.0009 |
| KO SED vs KO FEX | -14.50 | 2.871 | 0.0246 |

**Female Tendinopathy Scores Multiple Comparisons**

| Dunn’s Multiple Comparison | Mean Rank | Z-Value | Adjust P Value |
| --- | --- | --- | --- |
| WT SED vs WT FEX | -4.057 | 0.9390 | >0.9999 |
| WT SED vs KO SED | -7.557 | 1.392 | 0.9836 |
| WT SED vs KO FEX | -5.682 | 1.433 | 0.9110 |
| WT FEX vs KO SED | 3.500 | 0.6147 | >0.9999 |
| WT FEX vs KO FEX | -1.625 | 0.3761 | >0.9999 |
| KO SED vs KO FEX | 1.875 | 0.3454 | >0.9999 |

**Female Patellar Enthesitis Scores Multiple Comparisons**

| Dunn’s Multiple Comparison | Mean Rank | Z-Value | Adjust P Value |
| --- | --- | --- | --- |
| WT SED vs WT FEX | -8.063 | 2.933 | 0.0201 |
| WT SED vs KO SED | 0.000 | 0.000 | >0.9999 |
| WT SED vs KO FEX | -1.550 | 0.5996 | >0.9999 |
| WT FEX vs KO SED | -8.063 | 2.013 | 0.2647 |
| WT FEX vs KO FEX | 6.513 | 2.321 | 0.1218 |
| KO SED vs KO FEX | -1.550 | 0.3980 | >0.9999 |
